# The Miniaturization of Cardiac Implantable Electronic Devices: Advances in Diagnostic and Therapeutic Modalities

**DOI:** 10.3390/mi10100633

**Published:** 2019-09-21

**Authors:** Richard G. Trohman, Henry D. Huang, Parikshit S. Sharma

**Affiliations:** Section of Electrophysiology, Arrhythmia and Pacemaker Services, Division of Cardiology, Department of Internal Medicine, Rush University Medical Center, Chicago, IL 60612, USA; Henry_D_Huang@rush.edu (H.D.H.); Parikshit_S_Sharma@rush.edu (P.S.S.)

**Keywords:** implantable cardioverter defibrillators, cardiac pacing, cardiac resynchronization therapy, implantable heart failure sensor, implantable loop recorder

## Abstract

The Fourth Industrial Revolution, characterized by an unprecedented fusion of technologies that is blurring the lines between the physical, digital, and biological spheres, continues the trend to manufacture ever smaller mechanical, optical and electronic products and devices. In this manuscript, we outline the way cardiac implantable electronic devices (CIEDs) have evolved into remarkably smaller units with greatly enhanced applicability and capabilities.

## 1. Introduction

The Fourth Industrial Revolution [1], characterized by an unprecedented fusion of technologies that is blurring the lines between the physical, digital, and biological spheres, continues the trend to manufacture ever smaller mechanical, optical and electronic products and devices [2]. In this manuscript, we outline the way cardiac implantable electronic devices (CIEDs) have evolved into remarkably smaller units with greatly enhanced applicability and capabilities.

## 2. Prevention of Sudden Death

The current annual incidence of sudden cardiac death in the United States is in the range of 180,000 to 450,000 per year [3,4]. Although the prevalence of malignant ventricular arrhythmias as the etiology has declined, they remain the most common cause of cardiac arrest [3]. Multiple studies have confirmed the preeminence of implantable cardioverter defibrillators as the treatment of choice for primary and secondary prevention of sudden cardiac death [5].

The first implantable defibrillator (AID) was large (289 g, 150 mL), required a median sternotomy to open to place patch electrodes and two screw-in sensing leads on the heart’s epicardial surface. The pulse generator had to be implanted subcutaneously in the abdominal region [6].

The earliest implantable defibrillators had no or very limited capabilities with respect to pacemaker function, stored telemetry, and arrhythmia discrimination ability [6]. Modern devices can perform virtually all the functions of a pacemaker, including cardiac resynchronization therapy pacing, storing large amounts of arrhythmia and other physiologic data, and have some ability to discriminate between supraventricular and ventricular tachycardias [7].

Modern implantable cardioverter defibrillator (ICD) systems are implanted under the skin of the upper chest area and have two basic elements, a pulse generator and 1–3 leads placed in the cardiac chambers or epicardially via the coronary sinus into a cardiac vein to pace the left ventricle. They are available as single-chamber (right ventricular lead only), dual-chamber (right atrial and right ventricular leads) and triple-chamber devices (right atrial, right ventricular and left ventricular leads) capable of bradycardia, anti-tachycardia (termination), and cardiac resynchronization (triple-chamber only) pacing [8].

The majority of an ICD pulse generator consists of the battery and a capacitor (the component that stores and delivers charges). Defibrillators must store energy to deliver lifesaving shocks. Developing capacitors which required a minimum of stored energy but still delivered enough energy for defibrillation without affecting the ICD service life was pivotal in size reduction [9].

Capacitors are electronic components that take advantage of the ability of electrical fields to reach across an insulator. They consist of two flat plates made from a conducting material, separated by a thin insulating material. When a battery is connected to the conducting plates, the battery voltage negative side pushes negative charges toward one plate. The positive battery voltage side simultaneously pulls electrons (negative charge) away from the second plate. The electric field that rapidly builds between plates permits current to flow. As the circuit’s negative plate fills with electrons, the electric field created pushes electrons away from the plate on the opposite side of the insulator toward the positive battery voltage side. When current flows, an excess of electrons builds up on the negative capacitor plate as the positive side develops an electron deficiency, creating a potential difference (voltage) between the capacitor’s plates. Current only flows briefly. As electrons accumulate on the negative plate and are depleted on the positive plate, the differences in charge between plates increases and the voltage between them increases. The voltage increases until the capacitor voltage is equal to the battery voltage. Once the voltages are equal, current flow ceases, and the capacitor is charged. When the capacitor has been charged, the battery may be disconnected and the voltage remains in the capacitor (does not depend on the battery for its continued presence). Thus capacitors are capable of storing change (a quality known as capacitance) [10].

Electrolytic capacitors can hold a massive electric charge in their tiny footprint [10]. Current implantable cardioverter defibrillators (ICDs) are minicomputers that and are small enough to fit in the palm of your hand. The newest devices weigh as little as 70 g at a volume of less than 40 mL and are less than a centimeter thick [11].

The subcutaneous implantable cardioverter defibrillator (S-ICD) was approved for use in the United States in 2012 [12]. Although innovative, it is clearly a step backward from miniaturization and enhanced capability. S-ICDs do not have the capability of providing bradycardia pacing, antitachycardia pacing or cardiac resynchronization therapy. Even the third-generation pulse generator (EMBLEM™ MRI S-ICD System, Boston Scientific, St. Paul, MN, USA) is reminiscent of the early, bulky ICD models. It weighs 130 g and its volume is 59.5 cm^3^. Its height, width and thickness are 69.1 mm, 83.1 mm, and 12.7 mm, respectively [13].

S-ICDs may be considered for: (1) Younger patients due to the expected subcutaneous lead longevity and a desire to avoid chronic transvenous leads; (2) ICD candidates without a current or anticipated need for pacing; (3) Patients at high risk for bacteremia, including those with end-stage renal disease on hemodialysis or with chronic indwelling endovascular catheters; (4) Patients with limited vascular access or prior transvenous ICD complications [12].

Inappropriate shocks, mainly resulting from oversensing, are a significant limitation of S-ICDs. Non-invasive reprogramming options are not always successful and device explantation may be required.

## 3. Advances in Implantable Cardioverter Defibrillator (ICD) Lead Technology

Older DF-1 leads consist of bifurcated (in a single-coil lead) or trifurcated (in a dual-coil lead) header connector pins. One pin is a pace-sense connector and the other(s) are high-voltage coil connectors. The connectors are joined in a yoke that incorporates them into one lead body. The distal end of the lead is implanted in the right ventricle. The pulse generator header may have three plugs for connector insertion (dual-coil lead, single-chamber ICD), four plugs (dual-coil lead, dual-chamber ICD), or five plugs (dual-coil lead, cardiac resynchronization therapy defibrillator). Altogether, large headers combined with the bi/trifurcated yoke result in an ICD system that is quite bulky [12].

The four-pole inline DF-4 connector system was endorsed by the Association for the Advancement of Medical Instrumentation in 2011 [14]. DF-4 connectors are designed to facilitate lead-to-device connection, minimize the risk of incorrect device connection, and reduce the bulk of the device. Unfortunately, these advantages are achieved at the expense of additional connectivity, which precludes strategies such as adding a subcutaneous or azygos vein coil to overcome the problem of high defibrillation energy requirements [14,15].

## 4. Pacing for Bradycardia

Cardiac pacemakers were invented in 1949. The original versions were bulky boxes plugged into walls for power [16]. In 1958, three developments paved the way for modern cardiac pacing. Furman introduced a transvenous electrode and successfully stimulated the right ventricle (RV) for 96 days. Medtronic (Minneapolis, MN, USA) developed a four-inch, battery-powered box that could be taped to a patients’ chest and Lillehei and Bakken reported efficacy of a battery-powered external pacemaker in 18 patients. The first wearable pacemaker weighed 283 g. Later that year, Senning and Elmqvist performed the first pacemaker implant using an epicardial lead [16,17,18]. Soon thereafter devices became small enough to be implanted internally, but the need for frequent recharging was problematic. Medtronic produced the first commercially implantable pacemaker technologies in 1960 [16]. Rate-adaptive pacing first appeared in the 1980s. Despite development of multiple sensor types, as well as blending of sensor technologies, there has been no significant observable symptom benefit or impact on clinical outcomes from different sensors or combination of sensors [19]. Magnetic Resonance Imaging (MRI) conditional devices have been developed over the last 10 years [16].

It is currently estimated that nearly one million patients worldwide receive conventional permanent transvenous cardiac pacemakers annually. Pacemakers are limited by device-related complications. Adverse events related to cardiac pacemakers occur in 10% of recipients [20]. Typically these events are related to the surgical pocket, pulse generator or transvenous lead(s). Leads are vulnerable to fracture, insulation failure or dislodgement and can also cause venous thrombosis/occlusion, tricuspid regurgitation, and cardiac perforation. Lead-related endocarditis is a significant concern, with mortality rates reported between 12–31% [21]. Pulse generators have been associated with pocket hematoma, skin erosion and infection.

Although pulse generators have grown smaller and leads are thinner, reduction in pacing lead size has been a mixed blessing. Smaller leads appear to be more likely to result in cardiac perforation [22]. Additionally, device infection is on the rise [23]. FINELINE II™ Sterox EZ Leads (Boston Scientific, Minneapolis, MN, USA) screw-in active-fixation leads, have been used worldwide since 2001. A mannitol coating surrounds the helix, facilitating easy passage through the great veins of the thorax. The mannitol melts in the cardiac chamber allowing helix fixation. Lead durability has been proven satisfactory [24]. Unfortunately, Fineline™ leads are at high-risk for disruption (severance) with traction and difficulties during lead extraction procedures have led many operators to avoid use of these leads.

In response to these concerns, two leadless cardiac pacemakers have been developed for patients requiring permanent ventricular pacing. Two leadless pacing systems have been available: the Micra Transcatheter Pacing system (Medtronic, Inc. Minneapolis, MN) and the Nanostim Leadless Cardiac Pacemaker (Abbott; subsidiary St. Jude Medical, St. Paul, MN, USA). Both systems provide right ventricular sensing, pacing, and rate responsiveness. While both of these pacing systems are delivered percutaneously via the femoral vein through a catheter delivery system, they differ with respect to size, fixation to the myocardium, and responsiveness. The Nanostim recently had two major recalls: The first due to premature battery failure and the second due to spontaneous detachment of the docking button (a feature designed to allow retrieval of the Nanostim). Abbott is maintaining a worldwide halt on implantations of the Nanostim leadless pacemaker after reports surfaced of problems with the device’s docking button [25]. Hence, the remainder of this discussion will focus on Micra.

**Figure 1:**
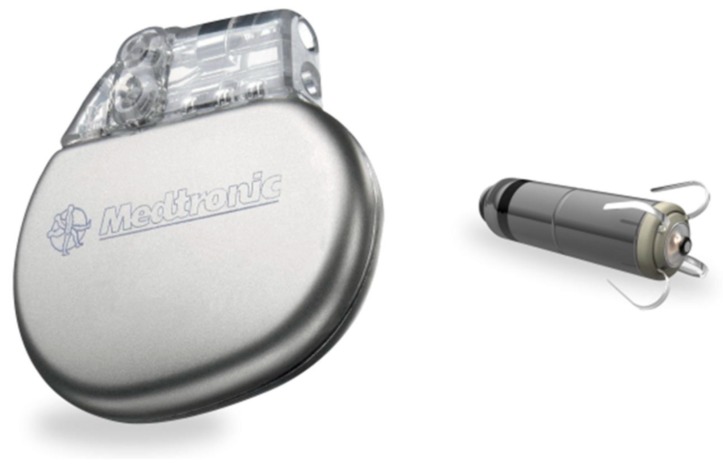
A typical dual-chamber pacemaker (**left**) and the Micra (**right**). Reproduced with permission from reference [26] and Medtronic, Inc.

Micra has a length of 25.9 mm, a volume of 0.8 mL and weighs 2 g. Its size has been compared to a “large vitamin” (Figure 1) [26]. A percutaneous transfemoral venous catheter-based approach is used to introduce the device into the right ventricle. Micra requires a 23 French (inner diameter)/27 French (outer diameter) sheath. Four nitonol tines are used to affix the device to the right ventricular septal myocardium. Multiple fluoroscopic views are used to ascertain fixation. At least two tines are required to assure stable fixation. Micra uses conventional radiofrequency communication to confirm acceptable pacing and sensing parameters and provides rate responsiveness using a 3-axis accelerometer. Micra is tethered to the introducer sheath and can be retrieved and repositioned if unacceptable pacing/sensing parameters are recorded. The introducer sheath and a goose neck snare can be used to remove a device that is no longer tethered.

In the Micra investigational device exemption prospective study, implantation was successful in 719 of 725 (99.2%) patients. Complications occurred in 3.4% of patients, including cardiac perforation (1.5%), vascular complications (0.7%), venous thromboembolism (0.3%), and increased pacing thresholds (0.3%). There were no device dislodgements. The trial included a pre-specified historical cohort of patients implanted with a single-lead transvenous permanent pacemaker. Micra implantation was associated with a 48% reduction in major complications compared to the transvenous permanent pacemaker group [27].

Despite these encouraging results, the downside of chronic right ventricular pacing (mechanical dyssynchrony leading to heart failure) limits the current use of leadless pacing. We await development of leadless VDD systems, dual-chamber systems and possibilities for cardiac resynchronization therapy to facilitate the expansion of leadless pacing to a broader group of patients.

## 5. Pacing to Treat Heart Failure

Cardiac resynchronization therapy (CRT) is applicable to 25–30% of patients with symptomatic systolic heart failure. Patients with left bundle branch block (LBBB) and QRS duration ≥ 150 ms are seen to benefit the most from CRT. Current evidence suggests that approximately 30% of the patients who are selected for CRT do not respond to this therapy [28]. However, there is a lack of standard definitions for CRT response and the spectrum of CRT response and reported response rates vary widely depending on metrics used and whether a placebo effect is considered [28].

**Figure 2:**
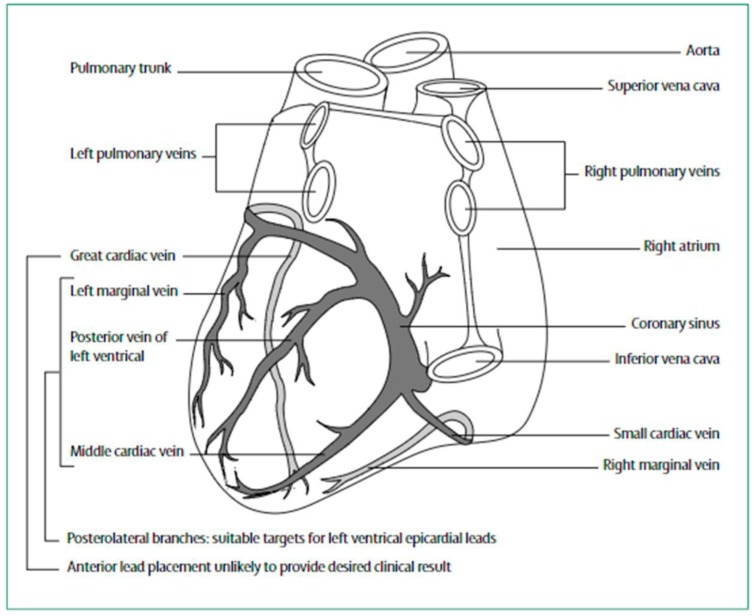
Diagrammatic presentation of heart showing positions for coronary venous lead placement. Reproduced with permission from reference [29].

CRT may be employed in association with bradycardia pacing (CRT-P) or an implantable cardioverter defibrillator (CRT-D). CRT is usually achieved via biventricular pacing. Right ventricular leads are placed via the great veins of the thorax. Left ventricular leads are also advanced via the great veins of the thorax and ultimately positioned in a branch of the coronary sinus (Figure 2) [29].

**Figure 3:**
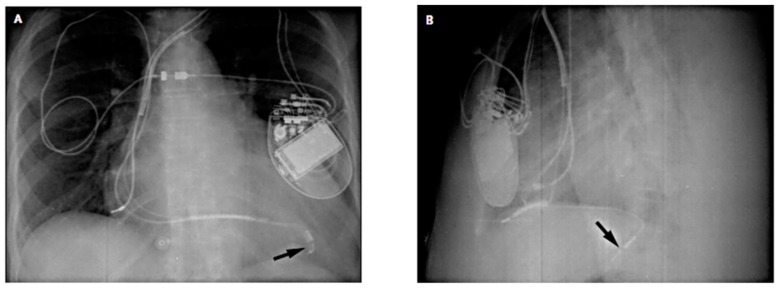
Chest radiographs showing lead placements. (**A**) Posteroanterior and (**B**) lateral chest radiographs showing tip of a large left ventricular lead (arrows) in a tributary of the middle cardiac vein. Despite proximity of the lead to the left hemidiaphragm, phrenic nerve stimulation did not take place. The right ventricular lead points anteriorly toward the rib cage. Reproduced with permission from reference [29].

Early attempts at biventricular pacing used leads that were not designed for cardiac venous placement. The Attain models 2187, 2188 (Medtronic, Inc., Minneapolis, MN) were stylet-driven and designed for atrial pacing. Lead lengths ranged from 58–85 cm and lead diameters ranged for 6.2–6.7 French. Although optimal outcomes could be achieved (Figure 3) [29], manipulation into small branches was frequently impossible. The subsequent development of novel narrow diameter leads easily advanced over guidewires has become the mainstream method for cardiac resynchronization therapy. The characteristics of state of the art left ventricular leads are summarized in Table 1.

A multi-center trial recently demonstrated that cardiac resynchronization can be achieved at least as well as biventricular pacing by substituting His bundle pacing (HBP) for cardiac venous pacing [30] (Medtronic, Inc., Minneapolis, MN). HBP is usually performed using the Select Secure (model 3830, 4 FR, 69 cm, Medtronic, Inc., Minneapolis, MN) pacing lead delivered through a fixed curve sheath (C315 HIS, Medtronic, Inc., Minneapolis, MN).

## 6. Pressure Monitoring to Manage Heart Failure

An estimated 57.4 million heart-failure-associated admissions occurred during the years 2001–2014. Although primary HF admission declined by an average annual rate of 3%, heart failure remains a major public health burden worldwide [31]. Additionally, hospital readmissions remain a continued challenge in the management of the heart-failure patients. Although small gains have been made over the past 5–7 years, over 20% of patients are still readmitted within 30 days and up to 50% are readmitted by 6 months [32].

Right heart catheterization remains an important diagnostic tool in cardiology’s armamentarium, providing direct hemodynamic data to determine cardiac output (CO), evaluating intracardiac shunts, assessing valve dysfunction, diagnosing pulmonary hypertension, judging the effects of pharmacotherapy for heart failure, and evaluating patients prior to heart and/or lung transplantation [33].

In the 1980s, development of balloon flotation catheters which could be left in place for prolonged periods resulted in a surge in right heart catheterization in critical care units. Current standard thermodilution catheterization have 6–7.5 French. diameters and are 110 cm in length [34]. Unfortunately, pulmonary arterial catheterzation-guided management was associated with increased mortality and length of intensive care unit stay and led to a substantial decline in their use [33].

In recent years, improvements in the diagnostic power and availability of non-invasive cardiac imaging modalities, in addition to evidence of potential harm associated with pulmonary artery catheterization in patients in critical care, have resulted in an additional decline in right heart catheterization [33].

The public health and financial burden associated with heart failure has spurred efforts to detect early markers of clinical deterioration. A variety of implantable sensors have been designed to create opportunities for preemptive intervention to facilitate better heart failure care [35].

Sensors incorporated into CRT systems have been used to monitor hemodynamic, biochemical and electrical parameters. Piezoelectric sensors have been added to specialized right ventricular leads to monitor right ventricular pressure, peak endocardial acceleration and mixed venous oxygen saturation. The requirement for specialized leads with risks of lead-related complications has limited the applicability of these options [35]. The HeartPOD, a sensor lead placed at the inter-atrial septum and attached to a coil antenna used to measure left atrial pressure, was studied in the LAPTOP-HF outcomes trial. This trial was terminated early due to the perception of excess of implant-related complications [35,36,37,38].

Measurement of heart rate variability is not practical during atrial pacing or atrial tachycardias and may be altered by the use of cardiovascular medications [35]. Monitoring physical activity with accelerometers is a potentially useful adjunct, but not a viable option for prompt intervention. Intrathoracic impedance measurements may lack sensitivity and/or specificity in the presence of pleural effusion or concomitant pneumonia [35]. Early detection (via CRT devices) and prompt treatment of atrial arrhythmias may limit their adverse effects in heart-failure patients.

The search for practical implantable pressure sensor has been vigorously pursued with research and development for many years [39]. Despite the reduction in size of pacemakers and ICDs, they remain relatively large and have limited battery lives. An ideal pressure sensor would eliminate bulky hermetic sealing [40].

Recent rapid improvement in microfabrication has enabled development of smaller implantable, highly accurate pressure sensors capable of chronic monitoring. Microelectro-mechanical systems (MEMS) have significant miniaturization advantages [40]. The design of these sensors is fairly simple consisting of a scaled-down deformable membrane (diaphragm) and electrodes placed on the top and bottom within a sealed cavity [40].

The CardioMEMS™ HF System (St. Jude Medical, Inc. (Abbott), Little Canada, MN, USA) is the first (and only) FDA-approved heart failure monitor proven to significantly reduce heart-failure hospital admissions and improve quality of life [41]. The system is a miniaturized, implantable wireless monitoring sensor that is placed percutaneously (via the femoral venous approach) in the pulmonary artery to directly measure pulmonary arterial pressure. The CardioMems PA sensor (St. Jude Medical, Inc. (Abbott), Little Canada, MN, USA) is delivered to the pulmonary artery via a catheter utilizing a 0.018” over-the-wire system. The sensor is attached to the distal catheter by a tethering release cord. The sensor measures 15 mm in length, 3.5 mm in width, and 2 mm in thickness. Two polytetrafluoroethylene-coated nitinol loops (each measuring 10 mm in diameter) keep the sensor in contact with the vessel walls after its release [42]. As noted, the sensor resides in a completely sealed capsule that uses microelectromechanical systems (MEMS) technology which allows measurement stability and energy efficiency.

The system is capacitive. Pressure deforms the membrane, changing the distance between the electrodes and increasing the capacitance across the electrodes. An LC electrical circuit (L = inductor; C = capacitor) stores energy and oscillates at the circuit’s resonant frequency [43]. The sensor is powered by radiofrequency (RF) energy (it does not require a battery) and made of materials chosen for biocompatibility, insensitivity to alterations in body chemistry or biology, and durability. It is designed to last the lifetime of the patient.

Once implanted, the sensor wirelessly sends pressure readings to the portable external patient electronic system. The electronic unit is turned on and reads pressure measurements wirelessly while the patient lies on a special pillow containing an antenna [44,45]. The electronic unit uses audible and visual signals, prompting the patient to press a button to initiate a reading.

The portable external electronic unit and the specialized pillow help complete a system that permits the implantable sensor size to be small. The system allows patients to transmit PA pressure data (to a secure website) from their homes to their health care providers, allowing for personalized and proactive management geared toward reducing the likelihood of hospitalization [45].

## 7. Minimally Invasive, Long-Term Heart Rhythm Monitoring

An implantable loop recorder (ILR) is a small device (1.2–6.5 mL in volume; less than the size of a chewing gum pack or USB memory stick [46,47]) implanted subcutaneously around the 4th intercostal space to the left of the sternum. These single-lead, electrocardiographic monitoring devices are used for diagnosis in patients with unexplained recurrent palpitations or syncope, for long-term monitoring in patients at risk for or with known atrial fibrillation (AF), cryptogenic stroke, for risk stratification in patients who have sustained a myocardial infarction and for individuals with certain genetic disorders. ILRs (with nearly 3 years of battery life) have a significantly greater diagnostic yield than 24-h Holter, 30-day event, or 30-day mobile cardiovascular telemetry monitors [46]. ILRs are exclusively diagnostic devices.

ILRs are leadless, have self-contained electrodes and solid-state loop memory capable of recording and storage of bipolar electrocardiogram (ECG) recordings when activated by a patient or bystander during symptomatic episodes [48]. The BioMonitor2 and BioMonitor3 (Biotronik, Lake Oswego, OR, USA) insertable cardiac monitors have antennae that are added to increase detection sensitivity. The antennae increase the device length (see Table 2 below). Each device primarily relies upon R-wave (ventricular activity) sensing. These devices can transmit data transtelephonically to a physician’s office for review. Device interrogation may also take place using individual manufacturers’ programmers.

Although ILRs have significant strengths compared to noninvasive monitoring devices, they also have several important limitations. These include oversensing, undersensing and a propensity for false-positive AF detection. False-positive episodes may be related to an irregular sinus rhythm, noise in the recording, ventricular and/or atrial ectopy [48].

## 8. Conclusions

Miniaturization has dramatically reduced the size of cardiac pacemaker and implantable defibrillator systems, while simultaneously facilitating advances in their therapeutic capabilities.

Additional advances in leadless pacing are likely to revolutionize the field in the near future. Smaller, more maneuverable leads have made cardiac resynchronization therapy for heart failure a practical, mainstream technique. Implantable monitoring for heart-failure recipients promises to reduce readmission rates and facilitate ongoing assessment of various pharmacological therapeutic interventions. Implantable loop recorders extend our ability to find the cause of unexplained syncope, define the etiology of infrequent palpitations and unveil occult atrial fibrillation as a mechanism of cryptogenic stroke. Smaller, faster devices with even greater capabilities hold great promise to help patients live longer and better electrically.

## Figures and Tables

**Table 1:** Cardiac venous (LV) leads sizes.

Manufacture	Prodtct Line	Lead Body Diameter	Lead Length Range
Abbott	Quartet^TM^	4.7 F	75–92 cm
Medtronic	Attain^TM^Performa^TM^	5.3 F	78–88 cm
Boston Scientific	Acuity^TM^X4	5.2 F	86–95 cm
Biotronik	Sentus^®^	4.8 F	77–97 cm

**Table 2:** Implantable loop recorder (ILR) sizes.

	Variable	Biotronik BioMonitor 2	Biotronik BioMonitor 3	Medtronic Reveal LINQ	St. Jude Medical Confirm
Size					
Length (mm)		88.4	77.5	44.8	56.3
Width (mm)		15.2	8.6	7.2	18.5
Thickness (mm)		6.2	4.6	4.0	8.0
Volume (cm^3^)		5.0	1.9	1.2	6.5
